# Behavioral and cognitive decline are observable in the aging house cricket (Acheta domesticus)

**DOI:** 10.1093/geroni/igaf122.3330

**Published:** 2025-12-31

**Authors:** Gerald Liao, Swastik Singh, Sherwin Dai, Elizabeth Bae, Warren Ladiges

**Affiliations:** University of Washington, Seattle, Washington, United States; University of Washington, Seattle, Washington, United States; University of Washington, Seattle, Washington, United States; University of Washington, Seattle, Washington, United States; University of Washington, Seattle, Washington, United States

## Abstract

Understanding the mechanisms of aging is critical for developing interventions against age-related diseases. While traditional mammalian models provide valuable insights, they are often limited by ethical and financial constraints. Alternative models, such as invertebrates, offer promising avenues for aging research. Here, we investigate the house cricket (Acheta domesticus) as a novel model organism for studying age-related behavioral and cognitive decline. We hypothesize that crickets, like humans and mice, exhibit progressive deterioration in locomotion, exploratory behavior, and cognition with age. Using adult (6-weeks), mid-age (8-weeks), and geriatric (>10-weeks) cohorts, we conducted a series of behavioral assays. In an open-field test, older crickets demonstrated reduced exploration, slower locomotion, and increased periphery preference. A modified treadmill assay revealed declines in maximum jumping distance, running speed, and endurance. Cognitive function was assessed using an aversive-learning escape paradigm, where crickets learned to associate cinnamon scent with a reward and vanilla scent with a punishment. Geriatric crickets exhibited impaired learning, though no differences were observed in short-term memory. Interestingly, mid-age crickets outperformed other groups in long-term memory. Additionally, adult and geriatric crickets reached decision arms faster than mid-age counterparts, with adults making the quickest choices. A scent preference test revealed that only adults demonstrated a significant preference for vanilla. These findings suggest that age-related phenotypic changes in house crickets are quantifiable through behavioral assays, supporting their potential as a scalable, cost-effective model for aging research. Further exploration of molecular and physiological markers could offer cross-species insights into the biology of aging.

